# Influence of Thiol-Functionalized Polysilsesquioxane/Phosphorus Flame-Retardant Blends on the Flammability and Thermal, Mechanical, and Volatile Organic Compound (VOC) Emission Properties of Epoxy Resins

**DOI:** 10.3390/polym16060842

**Published:** 2024-03-19

**Authors:** Young-Hun Kim, Jeong Ju Baek, Ki Cheol Chang, Ho Sun Lim, Myung-Seok Choi, Won-Gun Koh, Gyojic Shin

**Affiliations:** 1 Green Circulation R&D Department, Research Institute of Sustainable Development Technology, Korea Institute of Industrials Technology (KITECH), Yangdaegiro-gil 89, Ipjang-myeon, Cheonan-si 31056, Republic of Korea; kimyh@kitech.re.kr (Y.-H.K.); baekjj@kitech.re.kr (J.J.B.); kcchang@kitech.re.kr (K.C.C.); 2 Department of Chemical and Biomolecular Engineering, Yonsei University, Yonsei-ro 50, Seodaemun-gu, Seoul 03722, Republic of Korea; 3 Department of Chemical and Biological Engineering, Institute of Advanced Materials and System, Sookmyung Women’s University, Seoul 04310, Republic of Korea; limhs@sookmyung.ac.kr; 4 Division of Chemical Engineering, Konkuk University, 120 Neungdong-ro, Gwangjin-gu, Seoul 05029, Republic of Korea; mchoi@konkuk.ac.kr

**Keywords:** epoxy resin, polysilsesquioxane, residual char, flame retardant, cooperative effects

## Abstract

In this study, thiol-functionalized ladder-like polysesquioxanes end-capped with methyl and phenyl groups were synthesized via a simple sol-gel method and characterized through gel permeation chromatography (GPC), Fourier transform infrared spectroscopy (FTIR), nuclear magnetic resonance (NMR), and thermogravimetric analysis (TGA). Additionally, epoxy blends of different formulations were prepared. Their structural, flame-retardant, thermal, and mechanical properties, as well as volatile organic compound (VOC) emissions, were determined using differential scanning calorimetry (DSC), dynamic mechanical analysis (DMA), TGA, scanning electron microscopy (SEM), limiting oxygen index (LOI), cone calorimetry, and a VOC analyzer. Compared to epoxy blends with flame retardants containing elemental phosphorus alone, those with flame retardants containing elemental phosphorus combined with silicon and sulfur exhibited superior thermal, flame-retardant, and mechanical properties with low VOC emissions. SEM of the residual char revealed a dense and continuous morphology without holes or cracks. In particular, LOI values for the combustion of methyl and phenyl end-capped polysilsesquioxane mixtures were 32.3 and 33.7, respectively, compared to 28.4% of the LOI value for the blends containing only phosphorus compounds. The silicon–sulfur–phosphorus-containing blends displayed reduced flammability concerning the blends using a flame retardant containing only phosphorus. This reflects the cooperative effects of various flame-retardant moieties.

## 1. Introduction

Epoxy resins (EPs) are important thermoset materials that are extensively used in chemical, electrical, transport, and defense industries [1,2,3,4]. These materials can be used in various applications, such as coatings, adhesives, electrical and electronic parts, fiber reinforced, optical fiber, and flame retardant material, because of their excellent chemical resistance, adhesive strength, mechanical strength, electrical insulation, thermal stability, dimensional stability, and cost-effectiveness [5,6,7,8,9,10,11]. However, EPs are highly flammable materials with a limiting oxygen index (LOI) for combustion of ≤19.8 [12,13]. Moreover, application is limited by the production of large amounts of smoke and toxic gases during combustion [14,15]. Therefore, for most applications, EPs need to be modified by adding flame retardants [16]. Flame retardants include inorganic materials like metal hydrates [17], halogen-based materials containing bromine or chlorine [18], phosphorus-based materials centered on phosphoric acid esters [19], and nitrogen-based materials, like melamine cyanurate [20]. Recent developments include biomaterial wood composites that exhibit flame retardancy and biomimetic properties [21].

Two primary methods exist for enhancing the flame retardancy of EPs. The first involves the incorporation of brominated bisphenol A. The second entails blending flame retardant additives into the residual matrix, which is the most common process. However, halogenated flame retardants can produce toxic volatile dioxins from the polymer matrix during thermal decomposition in the event of an actual fire. Additionally, additive flame retardants are susceptible to migration [22,23].

Among various additive flame retardants, phosphorus-based compounds are the most effective alternatives to halogenated flame retardants. Because their presence provides excellent flame retardancy, and they do not promote the generation of toxic gases during combustion [24,25]. Furthermore, in contrast to other additive flame retardants prone to migration from the polymer matrix, phosphorus compounds typically exhibit good compatibility with polymers, preventing delamination [26].

Notably, the acidic thermal decomposition products in phosphorus compounds exert a strong dehydration effect, promoting cationic crosslinking and the formation of a char layer at the polymer surface [27,28].

This char layer acts as a physical barrier, insulating the polymer materials from further heat and flame exposure, slowing down its thermal decomposition, and suppressing the release of flammable gases that could feed the fire [29]. Currently, Diethyl (hydroxymethyl) phosphonate (DEHMP), a phosphorus compound with excellent compatibility with various polymers, is attracting interest as a potential flame retardant for polymer-based materials, such as epoxy resins [30]. However, adding flame retardants may reduce the thermal or mechanical properties of resins. Therefore, in addition to providing sufficient flame retardancy, minimizing resin property degradation is essential for tuning a flame-retardant additive [31].

Functionalized polysilsesquioxanes are widely regarded as a new generation of high-performance materials and hybrid organic–inorganic structures [32]. Methods for the synthesis of silsesquioxanes include sol-gel [33], hydrosilylation [34], ring-opening polymerization [35], and step-growth polymerization [36]. The sol-gel method is the most popular synthesis method because it provides a uniform environment for hydrolysis and condensation reactions and relatively mild reaction conditions, making it compatible with various organosilane precursors and functional groups, and allowing the synthesis of organic-inorganic hybrids [37].

Siloxanes are widely used as silicon-based flame retardants because they provide polymer blends with excellent mechanical properties, high thermal stability, and superior flame retardancy, even in small amounts [38]. Recently, much attention has been paid to flame retardants containing silicon, phosphorus, and sulfur [39]. Because of the cooperative effects of phosphorus–silicon and sulfur, compounds containing all these elements have been found to have excellent flame-retardant performance [40]. They work together to form a thermally stable carbon-residue surface, blocking heat and mass transfer during polymer decomposition.

Additive manufacturing, mainly 3D printing, represents a significant application area for epoxy resins. Recent studies have explored DGEBA epoxy blends containing various functional additives, including mixtures with photo-curable acrylic resin for 3D printing carbon fiber composites [41,42,43].

However, the issue of VOC emissions during 3D printing remains a concern. Minimizing VOC emissions from the epoxy resins used in the thermal curing process of 3D printing is a viable solution [44,45]. The thermal, mechanical, and flame retardant properties of epoxy systems containing phosphorus, silicon, and sulfur have been investigated [46,47]. However, the impact of these additives on VOC emissions during combustion has not been examined. This study aims to manufacture an epoxy blend that increases mechanical properties and flame resistance while minimizing VOC emissions. It also proposes a high-functionality epoxy blend that alleviates the issue of VOC emissions during the thermosetting of epoxy used in additive-manufacturing 3D printing.

Polysilsesquioxanes with a ladder structure, end-capped with CH_3_ and phenyl groups, respectively, have been synthesized for potential flame-retardant applications. They were characterized using gel permeation chromatography (GPC), Fourier transform infrared spectroscopy (FTIR), nuclear magnetic resonance (NMR), and TGA. Additionally, epoxy blends with different formulations were prepared for comparison with the properties of blends containing phosphorus-only flame retardants. The structure, flame retardancy, thermal, mechanical, and VOC emission properties of the cured epoxy blends were assessed using differential scanning calorimetry (DSC), a universal testing machine (UTM), dynamic mechanical analysis (DMA), thermogravimetric analysis (TGA), cone calorimetry, scanning electron microscopy (SEM), LOI determination, and a VOC analyzer.

## 2. Experimental

### 2.1. Materials

For thermal curing, the diglycidyl ether of bisphenol A (DGBEA, YD-128) with an epoxy equivalent weight of 172–176 g/eq was purchased from Kukdo Chemical (Seoul, Republic of Korea). It was vacuum-dried at 80 °C for 3 h and then stored in a dryer for use. m-Xylylenediamine (m-XDA, 99%), supplied by Sigma-Aldrich (Saint Louis, MO, USA), is an aromatic amine in the liquid state, chosen for its excellent compatibility with DGEBA at room temperature. It facilitates the formation of an efficient curing system with epoxy resin. Diethyl (hydroxymethyl) phosphonate (DEHMP, 97%), supplied by TCI (Tokyo, Japan), is a liquid at room temperature, making it easily mixable with various additives, including epoxy resins. It was chosen for its ability to enhance flame retardancy without adversely affecting the material’s processing characteristics. TCI Chemicals supplied materials such as (3-Mercaptopropyl)trimethoxysilane(95%), Methoxytrimethylsilane (MTMS, 98%), and Methoxytriphenylsilane (MTPS, 98%) that are suitable for forming polysilsesquioxanes through the sol-gel process. All materials were used as received, without any additional purification.

### 2.2. Synthesis of Thiol-Functionalized Ladder-like Polysilsesquioxane End-Capped with Methyl Groups (TFLPM)

First, 30 g of solvent (tetrahydrofuran [THF]) and 5 g of (3-mercaptopropyl) trimethoxysilane monomer (25.46 mmol) were added to a 250-mL two-necked flask and stirred using a magnetic stirrer. Second, a 0.05N sodium hydroxide aqueous solution (1.5 g) was added to the mixture. Then, the solution was heated at 50 °C for 16 h under a nitrogen atmosphere. After the reaction, MTMS (0.58 g, 5.57 mmol) and 0.91 g of hydrochloric acid (36.5% aqueous solution, 25 mmol) were added and stirred at 50 °C for 8 h. After removing the solvent from the reaction mixture, it was washed several times with dichloromethane and distilled water. Then, dichloromethane and impurities were removed using a rotary evaporator and dried in a vacuum oven at 100 °C overnight.

TFLPM: ^1^H NMR (300 MHz, CDCl_3_, ppm): *δ* = 0.07–0.20 (m, Si–CH_3_), 0.71–0.84 (s, Si–CH_2_), 1.25–1.48 (m, Si–CH_2_–CH_2_–CH_2_–SH), 1.63–1.79 (d, Si–CH_2_–CH_2_), 2.49–2.66 (d, Si–CH_2_–CH_2_–CH_2_), ^13^C NMR (100.62 MHz, CDCl_3_, ppm): *δ* = 1.34 (Si–CH_3_), 11.18 (Si–CH_2_), 27.77 (Si–CH_2_–CH_2_–CH_2_–SH). Yield: 78%.

### 2.3. Synthesis of Thiol-Functionalized Ladder-like Polysilsesquioxane End-Capped with Phenyl Groups (TFLPP)

**Scheme 1:**
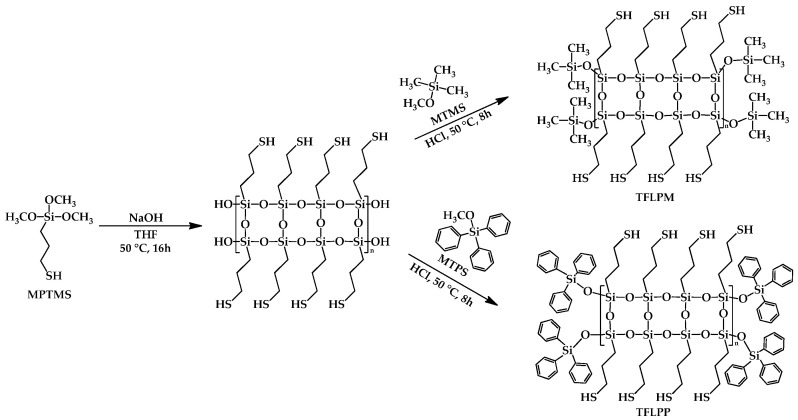
Synthesis scheme of TFLPM and TFLPP.

First, 30 g of solvent (THF) and 5 g of (3-mercaptopropyl) trimethoxysilane monomer (25.46 mmol) were added to a 250 mL two-necked flask and stirred using a magnetic stirrer. Second, a 0.05 N sodium hydroxide aqueous solution (1.5 g) was added to the mixture. Then, the solution was heated at 50 °C for 16 h under a nitrogen atmosphere. After the reaction, MTPS (0.52 g, 1.79 mmol) and 1.56 g of hydrochloric acid (36.5% aqueous solution, 43 mmol) were added and stirred at 50 °C for 8 h. After removing the solvent from the reaction mixture, it was washed several times with dichloromethane and distilled water. Then, dichloromethane and impurities were removed using a rotary evaporator and dried in a vacuum oven at 100 °C overnight. The synthesis of TLPM and TFLPP is shown in Scheme 1.

TFLPP: ^1^H NMR (300 MHz, CDCl_3_, ppm): *δ* = 0.71–0.88 (s, Si–CH_2_), 1.25–1.51 (m, Si–CH_2_–CH_2_–CH_2_–SH), 1.61–1.82 (d, Si–CH_2_–CH_2_), 2.45–2.68 (d, Si–CH_2_–CH_2_–CH_2_), 7.28–7.67 (m, Si–PH). ^13^C NMR (100.62 MHz, CDCl_3_, ppm): *δ* = 11.75 (Si–CH_2_), 27.31 (Si–CH_2_–CH_2_–CH_2_–SH), 127.82 (Ortho-C), 130.28 (Para-C), 134.06 (Meta-C). Yield: 72%.

### 2.4. Preparation of Epoxy Blends (EP, EP-DP5, EP-TM5-DP5, and EP-TP5-DP5)

Flame retardants (TFLPM, TFLPP, and DEHMP in a total weight ratio of 10 wt%) were added into DGBEA, with the weight of the curing agent m-XDA fixed at 10 wt%. The mixture was stirred magnetically at 400 rpm for 30 min, followed by curing at 130 °C for 1 h and at 150 °C for an additional 1 h. The formulations for the flame-retardant EP specimens are detailed in Table 1.

### 2.5. Characterization of TFLPM, TFLPP, and Epoxy Blends

TFLPM and TFLPP were structurally analyzed using a Spectrum-400 FTIR spectrometer (Perkin Elmer, Waltham, MA, USA) that conducted 100 scans in the wavenumber range of 650–4000 cm^−1^. The ATR crystals at room temperature received the samples (10 μm) through dropping. All spectra were adjusted through CO_2_ reduction, noise removal, and baseline fitting. ^1^H, ^13^C, and ^29^Si NMR spectra were performed with CDCl_3_ as the solvent and using 300-MHz NMR equipment (Bruker, Billerica, MA, USA, Avance 300) at room temperature.

The molecular weights of TFLPM and TFLPP were determined through size exclusion chromatography using an EcoSEC HLC-8320 GPC from TOSOH Corporation (Tokyo, Japan). A 0.15% solution (wt/vol) of TFLPM or TFLPP in THF was injected into the GPC system. Separation utilized a combination of Guard Super MP (HZ)-M+2 and TSK Gel Super-multipore HZ-M columns (150 mm × 4.6 mm, 3 μm). The mobile phase comprised high-performance liquid chromatography-grade THF, flowing at a rate of 0.45 mL/min. The column temperature was set at 40 °C. The GPC system was calibrated with a range of thin polystyrene molecular weight standards: 580, 2980, 9960, 30,230, 69,650, 128,600, 325,600, and 660,500 Da.

The thermal stability of the samples was determined using a TGA-4000 thermal analyzer (Perkin Elmer, Waltham, MA, USA). Here, 5–10 mg samples were heated in a nitrogen environment at a rate of 10 °C/min from 50 °C to 800 °C.

A DSC (Perkin-Elmer DSC-8500, Waltham, MA, USA) was used to investigate the thermal behavior of EP, EP-DP5, EP-TM5-DP5, and EP-TP5-DP5. A 5 to 10 g sample was placed in an aluminum pan for the DSC, and the experiment was conducted under a nitrogen atmosphere of 5 °C/min in the 30–200 °C range. The thermal stability and decomposition temperatures of the fully cured epoxy compound were measured by TGA using a Perkin Elmer Pyris 1 thermal analyzer. Samples weighing 5–10 mg were placed on a ceramic pan and heated in a nitrogen atmosphere to prevent oxidation. The heating process was performed at a constant rate of 10 °C/min from 50 °C to 800 °C. The thermogravimetric analysis (TGA) was carried out on TGA Q5000 IR thermal gravimetric analyzer (TA Instruments, New Castle, DE, USA). About 4–10 mg of epoxy resins was heated from room temperature to 800 °C under air or nitrogen purges. Cone calorimetry tests were conducted using the cone calorimeter (Fire Testing Technology, East Grinstead, UK) following ISO 5660 standard [48] procedures. Each specimen, with dimensions of 130 × 130 × 3 mm^3^, was prepared, mounted on aluminum foil, and irradiated horizontally at a heat flux of 35 kW m⁻^2^.

Thermomechanical properties were measured using DMA under the nitrogen atmosphere in tensile mode. The constant frequency was 1 Hz, and the heating temperature was from −50 °C to 200 °C. The dimension of the specimens was 25 mm × 25 mm. The total VOC (TVOC) emission from the samples was quantified using a thermal extractor (TE, Gerstel, Linthicum, MD, USA) with flow regulation (10–300 mL/min). The VOCs were released from the samples by carrier gas at a 134 mL/min flow rate and collected in an adsorption tube. A separate glass extraction tube was used for each 25 mg sample. The TE consisted of a controllable (room temperature) furnace that heated the glass tube (178 mm, diameter 13.6 mm) containing the sample. VOC analysis was performed according to ES 02603.1: “Methods for measurement of VOC emissions from interior and construction materials—Solid absorber tubes and GC-MS/FID method”. VOCs were extracted using a Tenax TA adsorption tube (Supelco, Bellefonte, PA, USA) and a micro-pump (MP-30, SIBATA, Saitama, Japan) under a steady flow of pure nitrogen gas. A total of 1 L of gas was sampled during the thermal extraction procedure at 25 °C ± 5 °C for 30 min. Individual calibration lines for the following compounds were used for the qualitative analysis: TVOC, benzene, toluene, ethylbenzene, *o*-, *m*-, *p*-xylene, and styrene. The TVOC concentration (µg/m^3^) was calculated using the toluene calibration curve for the total area of the chromatogram between *n*-hexane and *n*-hexadecane. The epoxy cure blends of LOI were determined using an LOI tester (FTT, Derby, UK) by making specimen dimensions 130 × 6.5 × 3.0 mm^3^ according to the ASTM 2863 standard [49]. After placing the specimen vertically in the LOI tester, a constant flow of high-purity nitrogen and oxygen was introduced from the bottom, and the combustion behavior was checked by igniting the leading edge of the specimen. SEM analysis was performed using Nova Nano FE-SEM 450 (FEI Company, Hillsboro, OR, USA).

The LOI value was calculated using the following Equation (1).

(1) $$\mathrm{LOI}=\frac{\left[O_{2}\right]}{\left(\left[O_{2}\right]+\left[N_{2}\right]\right)}\times 100,$$

where [O_2_] is the oxygen flow rate (L/min^−1^) and [N_2_] is the nitrogen flow rate (L/min^−1^). The flammability of epoxy-cured blends materials was measured following the ASTM 3801 standard [50] with a specimen size of 130 × 130 × 3 mm^3^, pretreatment at 23 °C ± 2 °C, and 50% ± 5% relative humidity for 48 h. A universal material testing machine model 5567 (universal testing machine, UTM, Instron, Norwood, MA, USA) was used to measure the tensile strength of the epoxy blends following ASTM D638 [51]. Tensile tests were performed at a 10 mm/min test speed, 10 tests were performed for each specimen, and the mean value was used. A lap shear test was performed to measure the mechanical properties of the specimen. The test specimen consisted of an aluminum plate with an area of 25.4 × 10 mm^2^ coated with a 0.2 mm thick epoxy adhesive layer. Tests were performed according to ASTM D1002 [52], using a model 5567 universal testing machine (Instron, Norwood, MA, USA) at a speed of 1.3 mm (0.05 in)/min. Before testing, specimens were cured for 3 h at 130 °C in an oven. Lap shear strength was calculated as an average of five samples for each configuration, with error bars representing one standard deviation.

## 3. Results and Discussion

### 3.1. Structural Analyses of TFLPM and TFLPP

**Figure 1:**
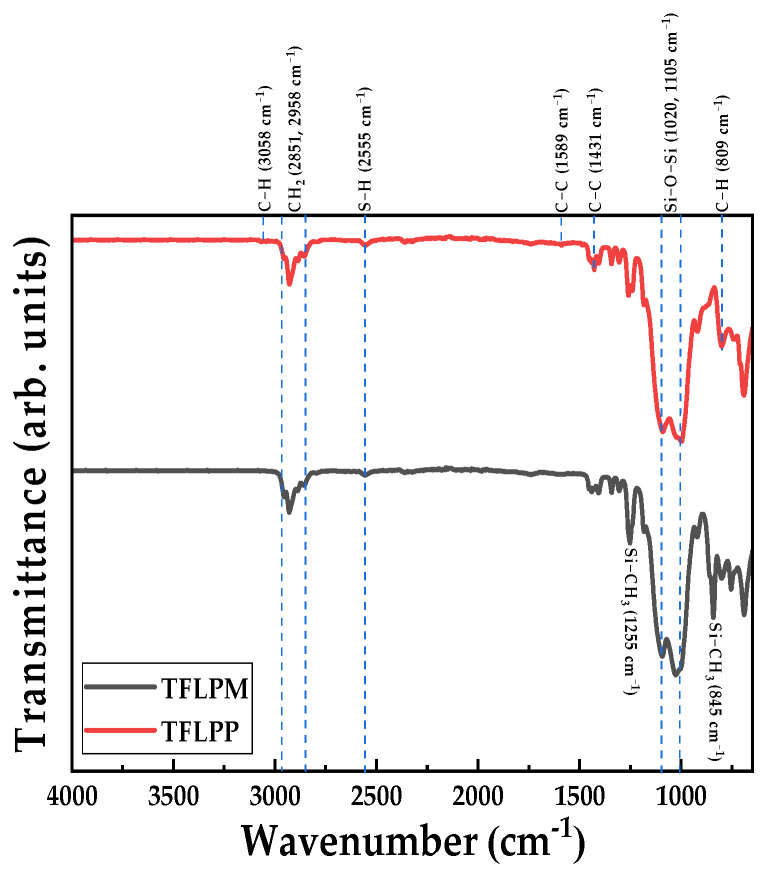
FTIR spectra of the synthesized TFLPM and TFLPP.

Figure 1 displays the FTIR spectra of TFLPM and TFLPP, highlighting several characteristic signals confirming the successful synthesis of the synthetic materials. The presence of sharp and intense signals at 845 and 1255 cm^−1^ in TFLPM corresponds to the bending vibrations of Si−CH_3_. These signals were detected with high intensity only in TFLPM. TFLPP also identified vibration absorption signals at 1431 and 1589 cm^−1^, which were the C−C stretching vibrations. The C−H stretching vibrations of the phenyl groups were detected at 809 and 3058 cm^−1^, respectively. In both TFLPM and TFLPP, the mercapto S−H stretching mode of the mercapto groups shows a weak signal at 2555 cm^−1^, and the absorption bands at 2851 and 2958 cm^−1^ were assigned to the alkane−CH_2_ groups. For ladder-structured polysilsesquioxanes, TFLPM and TFLPP exhibited characteristic horizontal and vertical silsesquioxane signals at 1020 and 1105 cm^−1^. In general, closed cage structures, such as polyhedral oligomeric silsesquioxane (POSS), display a single strong absorption signal near 1100 cm^−1^, while ladder-like structures around 1050 and 1150 cm^−1^ exhibit two absorption signals [53]. The TFLPM and TFLPP absorption signals exhibited a similar pattern to those observed in previous studies of ladder-like polysilsesquioxanes [54].

Structural analysis of NMR spectra for TFLPM and TFLPP is provided in the Supporting Information. Specifically, Figure S1a,b presents the ^1^H NMR spectra of TFLPM and TFLPP, respectively, while Figure S2a,b displays their corresponding ^13^C NMR spectra. Gel permeation chromatography (GPC) was employed to determine TFLPM and TFLPP molecular weights. Table 2 presents the results, indicating an average molecular weight of 3782 for TFLPM and 4682 for TFLPP, based on polystyrene calibration. The polydispersity indexes (PDIs) were measured as 1.78 and 1.95 for TFLPM and TFLPP, respectively. Figure S3a,b presents the Maldi-TOF MS spectra of TFLPM and TFLPP, respectively.

**Figure 2:**
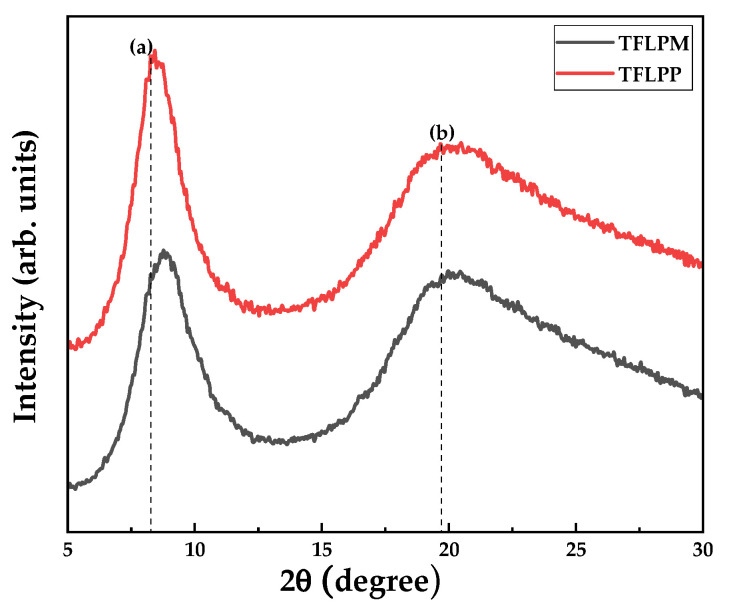
XRD pattern of TFLPM and TFLPP.

An XRD analysis was conducted to investigate the structures of TFPLPM and TFLPP, as presented in Figure 2. According to the literature [55], laddered polysilsesquioxanes typically manifest two characteristic diffraction signals. Two distinct diffraction signals were observed around 8° and 20°, corresponding to the distance between their organic groups (designated as a) and the length of their siloxane bond (Si-O-Si) (designated as b), respectively [56]. The dimensions corresponding to the a and b diffraction signals were calculated to be 14.1 and 4.1 Å for TFLPM and 14.7 and 4.4 Å for TFLPP.

### 3.2. Comparative Analysis of Thermal Characteristics of TFLPM and TFLPP

**Figure 3:**
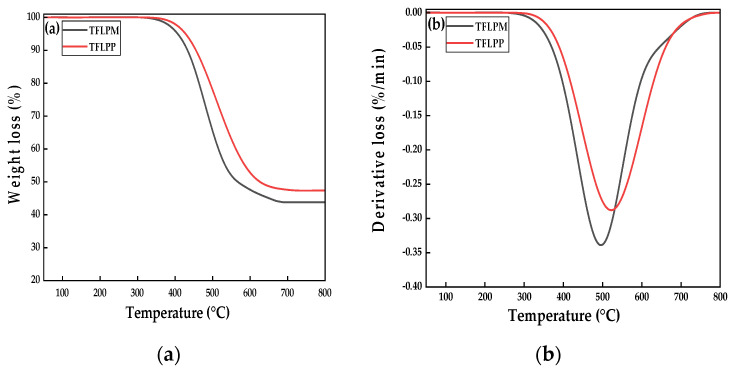
(**a**) TGA and (**b**) DTG curves of TFLPM and TFLPP under nitrogen atmospheres.

Figure 3a,b display the decomposition curves of TFLPM and TFLPP, respectively. As seen in Table 3, TFLPP exhibits a higher maximum decomposition temperature than TFLPM. This includes the decomposition temperature at a 5% weight loss (*T*_dec−5%_), the decomposition temperature at maximum weight loss (*T*_max_), thermal stability indices (A*, K*), and the integral procedural decomposition temperature (IPDT). The decomposition temperature at a 5% weight loss was 407.38 °C for TFLPM and 429.77 °C for TFLPP. Additionally, due to the formation of the polysilsesquioxane structure, the residual mass of TFLPM and TFLPP reached 43.82% and 47.53%, respectively.

Doyle’s integral procedural decomposition temperature (IPDT) provides a quantitative measure of thermal stability [57]. Calculated as a ratio of areas, IPDT remains consistent regardless of the number of decomposition steps observed in TGA analysis. A* is a simplified value that combines the residual mass and temperature and can be expressed as a ratio of the total area under the TGA thermal analysis curve and the residual mass. In this case, thermal stability was mainly influenced by initial decomposition temperature and residual mass. To calculate IPDT and assess the thermal stability of TFLPM and TFLPP, the area under the decomposition curve was integrated using Equation (2):

IPDT (°C) = A* ∙ K* (*T*_f_ − *T*_i_) + *T*_i_, (2)

where

A* is the area ratio of the total curve and total TGA thermo-gram ((S_1_ + S_2_)/(S_1_ + S_2_ + S_3_)),

K* is the coefficient of A* ((S_1_ + S_2_)/S_2_),

*T*_i_ is the initial experimental temperature (50 °C), and

*T*_f_ is the final experimental temperature (800 °C).

**Figure 4:**
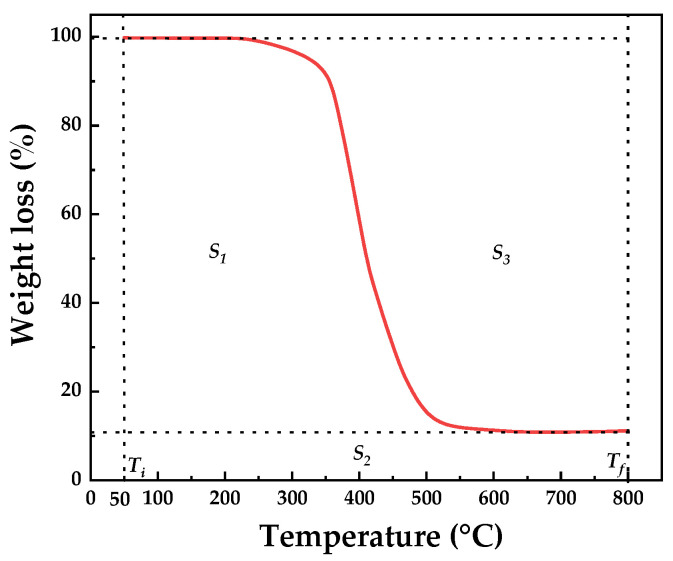
Schematic of S_1_, S_2_, and S_3_ for A* and K*.

Figure 4 presents a schematic for calculating IPDT. S_2_ is obtained by multiplying the residual amount by the temperature interval. S_1_ is the difference between the total area under the decomposition curve and S_2_. S_3_ represents the remaining area under the baseline. As shown in Table 3, both TFLPM and TFLPP exhibited excellent thermal stability and heat resistance, with IPDT values of 706.31 and 725.74 °C, respectively. The observed difference in thermal stability can be attributed to the varying molecular weights and properties of phenyl and methyl groups. Polymers with higher molecular weights generally exhibit greater resistance to high temperatures due to enhanced van der Waals intermolecular forces [58].

Additionally, the difference in thermal stability between silsesquioxanes with phenyl and methyl groups can be explained by the bond dissociation energies (BDEs) of Sp^2^ and Sp^3^ hybridized C-H bonds. Sp^2^ hybridized C-H bonds in aromatic rings, like phenyl groups, exhibit higher BDEs than Sp^3^ hybridized C-H bonds in methyl groups. This disparity is due to the Sp^2^ carbon atoms’ larger s-character (33%) and the resulting shorter, stronger bonds from the greater overlap between carbon and hydrogen s-orbitals, compared to the 25% s-character in Sp^3^ hybridized carbons [59,60]. Therefore, more energy is required to break Sp^2^ C-H bonds than Sp^3^ C-H bonds, making silsesquioxanes with phenyl groups less susceptible to bond cleavage under thermal conditions.

### 3.3. Comparative Analysis of the Thermal Stability of Epoxy Blends

**Figure 5:**
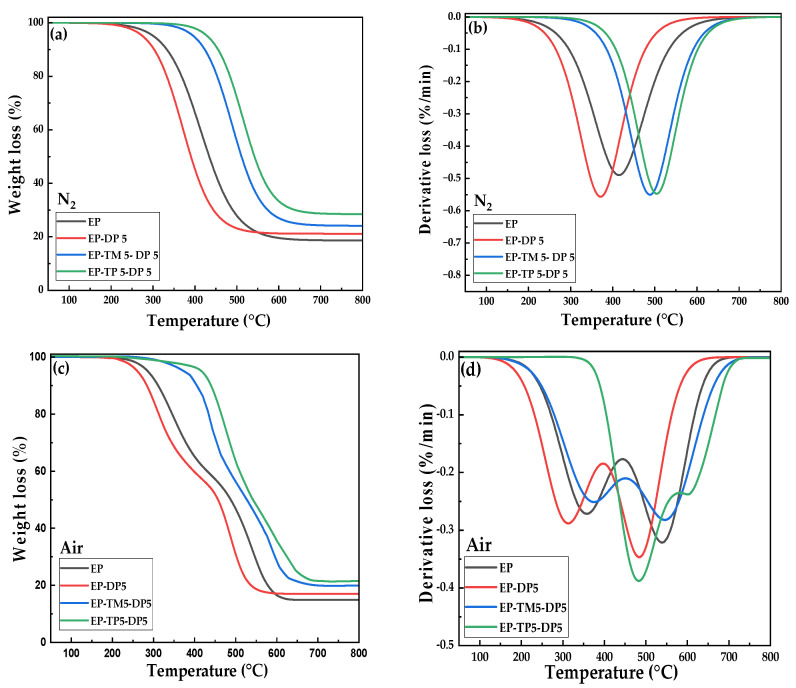
Thermogravimetric analysis (TGA) and derivative thermogravimetric (DTG) curves of epoxy blends in nitrogen (**a**,**b**) and air (**c**,**d**) atmospheres.

Figure 5a,b display the TGA and DTG curves of epoxy blends in a nitrogen atmosphere, while Figure 5c,d show the TGA and DTG curves of epoxy blends in an air atmosphere. Based on the thermal analysis, important thermal safety factors, temperature of decomposition at 5% weight loss (*T*_dec−5%_), the decomposition temperature at maximum weight-loss (*T*_max_), thermal stability indexes (A*, K*), and IPDT, were calculated and presented in Table 4.

Pure EP exhibited a decomposition temperature of 305.54 °C at a 5% weight loss in the nitrogen atmosphere, with a char yield of 18.63% at 800 °C. The char yield showed a linear increase to 21.26 (EP-DP5), 24.22 (EP-TM5-DP5), and 28.49% (EP-TP5-DP5) for the phosphorus-only and silicon–sulfur–phosphorus flame retardant-added blends, respectively. EP-DP5, containing only phosphorus flame retardant, showed a remarkable decrease in decomposition temperature (270.49 °C) at a 5% weight loss compared to pure EP. EP-DP5 with phosphorus flame retardant alone likely led to a plasticizing effect, reducing the epoxy’s crosslinking density and glass transition temperature, as evidenced by the decrease of temperature of decomposition at 5% weigh loss [61]. In particular, EP-TP5-DP5 displayed superior thermal stability and char yield due to its high phenyl content, which enhances molecular chain stiffness and improves thermal stability [62]. 

Under air conditions, the TGA curves for epoxy blends reveal a two-step degradation process: the decomposition of the epoxy chain followed by the decomposition between the char residue and oxygen [63]. Compared to nitrogen environments, the epoxy blends in air exhibit slightly lower *T*_dec−5%_ (°C), *T*_max_ (°C), and IPDT values. The observed thermal stability of the epoxy blends suggests their stability for applications even under oxidative conditions.

### 3.4. LOI Test and UL94-V Ratings for Epoxy Blends

The limited oxygen index (LOI) is a widely used metric to assess polymeric materials’ flammability and flame retardancy [64]. Materials exhibiting an LOI above 30% are categorized as flame retardants [65]. Such materials must also receive a UL 94 V-0 rating to be considered flame retardant [66]. Details regarding the UL 94 V test requirements can be found in Table S1.

**Figure 6:**
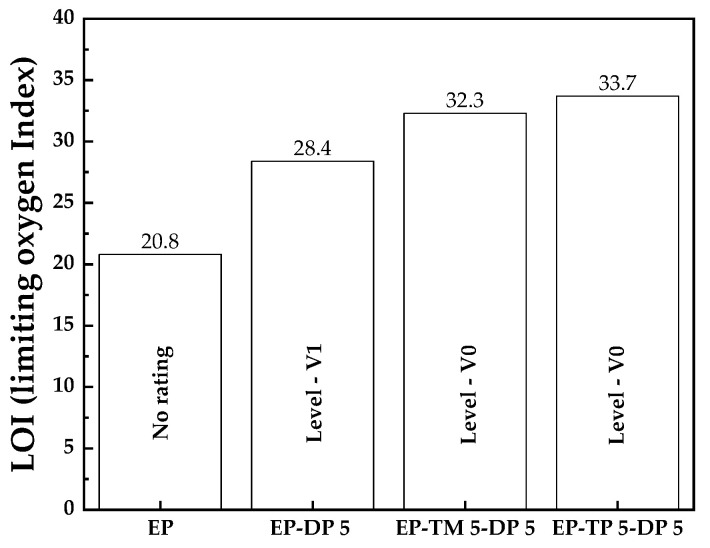
LOI and UL-94V test results for the cured epoxy blends.

The LOI and UL-94 V tests evaluated the flame retardancy of the EP system, with results presented in Figure 6. Pure EP exhibited high flammability, evidenced by a low LOI of 20.8% and an NR rating in UL-94 testing due to the melt-dropping phenomenon. EP-DP5 systems containing phosphorus flame retardants increased the LOI to 28.4% and achieved a UL-94 V-1 rating. Furthermore, the EP-TM5-DP5 and EP-TP5-DP5 systems, incorporating silicon, sulfur, and phosphorus, reached a UL-94 V-0 rating and achieved LOI values of 32.3 and 33.7%, respectively. These results suggest a cooperative effect within the EP-TM5-DP5 and EP-TP5-DP5 systems, which incorporate silicon, sulfur, and phosphorus flame retardants, leading to enhanced flame retardancy compared to systems containing only phosphorus additives.

### 3.5. Cone Calorimeter Test of Epoxy Blends

**Figure 7:**
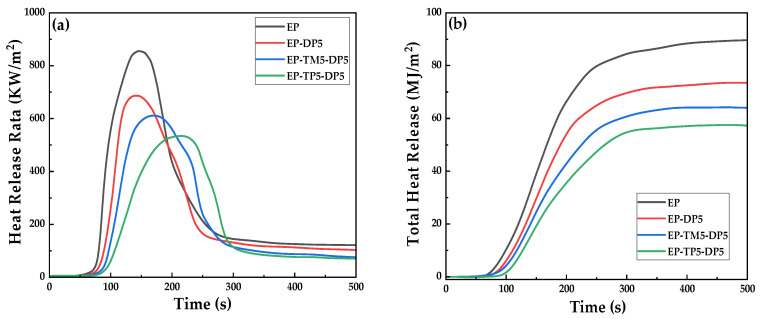
Cone calorimeter test. (**a**) HRR and (**b**) THR curves of epoxy blends.

The cone calorimetry test (CCT) is a widely adopted method for evaluating the flame retardancy performance of polymers. The test results directly correlate with the actual calorific value of combustion and offer important combustion parameters, making it one of the most advanced methods for combustion behavior assessment [67]. Figure 7 presents the characterization curves for heat release rate (HRR) and total heat release (THR). Cone calorimetry data (CCT), such as time to ignition (TTI), maximum HRR, and total THR, are listed in Table 5.

Figure 7a,b demonstrates a significantly lower heat release rate (HRR) and total heat release (THR) for EP blends compared to pure EP. Additionally, as shown in Table 5, the time to ignition (TTI) is significantly extended to 85 s for EP-TM-DP5 and 91 s for EP-TP5-DP5, compared to 73 s for pure epoxy. This extended TTI suggests a longer potential escape window for individuals in the event of a fire. These fire test results, therefore, demonstrate that the sulfur and phosphorus–silicon system combination effectively enhances the flame retardancy of EP.

### 3.6. Morphology of Epoxy Blends

**Figure 8:**
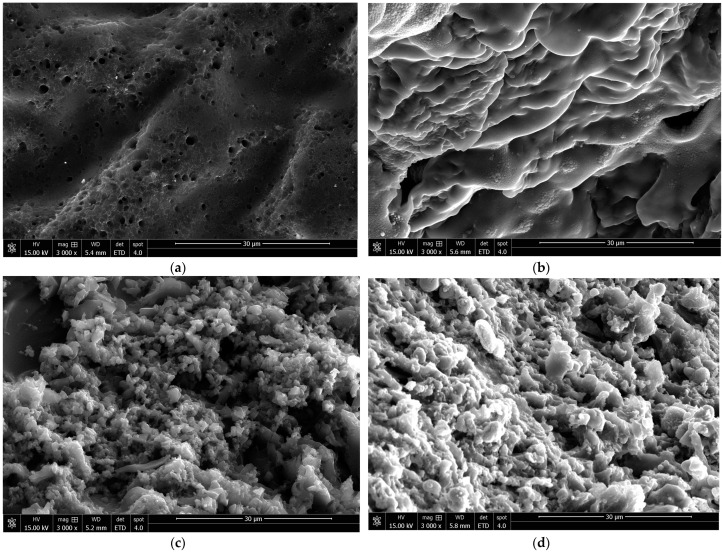
Residual char morphologies: (**a**) EP; (**b**) EP-DP5; (**c**) EP-TM5-DP5; and (**d**) EP-TP5-DP5.

Scanning electron microscopy (SEM) analysis was used to investigate the connection between the microstructure and flame retardancy of the EP system’s char layer after combustion [68,69]. The findings are presented in Figure 8a–d. Figure 8a reveals a discontinuous surface for the pure EP, characterized by numerous holes and identifiable cracks on the char residue. This structure is ineffective in preventing the migration of flammable decomposition volatiles and the penetration of oxygen and heat, leading to poor flame-retardant properties. Figure 8b demonstrates that incorporating a phosphorus-containing flame retardant results in a more continuous and dense char layer with fewer holes and more char residue, indicating improved flame retardancy. Figure 8c,d shows that adding a silicon–sulfur–phosphorus-containing flame retardant further enhances the char layer’s continuity and density, accompanied by increased char residue, improving the flame retardant performance.

Energy-dispersive X-ray spectroscopy (EDX) analysis determined the elemental composition of the epoxy blend char residue (C, O, N, Si, P, and S), as shown in Table 6. Compared to pure EP, both EP-DP5 (containing phosphorus) and the silicon- and phosphorus-containing blends, EP-TM5-DP5 and EP-TP5-DP5, exhibited an increased carbon weight percentage. This increase in EP-TM5-DP5 and EP-TP5-DP5 is attributed to the stabilizing effect of silica residue. Especially, EP-TP5-DP5, with the highest sulfur content, also displayed the highest carbon weight percentage. These results suggest that EP-TP5-DP5, due to its high sulfur content, releases relatively more SO_2_, a non-combustible gas that dilutes the atmosphere and enhances flame retardancy.

### 3.7. Comparative Analysis of the Thermomechanical Properties of Epoxy Blends

Quantifying internal structure and rheological changes during thermoset gelation remains a significant challenge. Therefore, dynamic viscoelasticity measurements are typically performed post-reaction. These measurements offer insight into physical and mechanical properties through rheological parameters like viscosity and dynamic moduli. Specifically, the storage modulus (G′) characterizes the elastic behavior, while the loss modulus (G″) reflects the viscous component. In particular, at the gelation point, the loss tangent becomes independent of frequency. Additionally, the point of overlap between G′ and G″ (tan δ = 1), as proposed by Tung and Dynes [70], can be determined as the gelation point.

(3) $$tan\delta =\left(\frac{G^{\prime \prime }}{G^{\prime }}\right)=tan\left(\frac{n\pi }{2}\right).$$

Both the loss modulus (G″) and loss tangent (tan δ) exhibit sensitivity to material molecular motion and transitions. As crosslinked epoxy molecules initiate micro-Brownian motion, both viscoelastic properties increase. This increase is reflected as a peak in the tan δ graph, signifying segmental diffusion and marking the gelation point. The corresponding temperature at this point is designated as the glass transition temperature.

**Figure 9:**
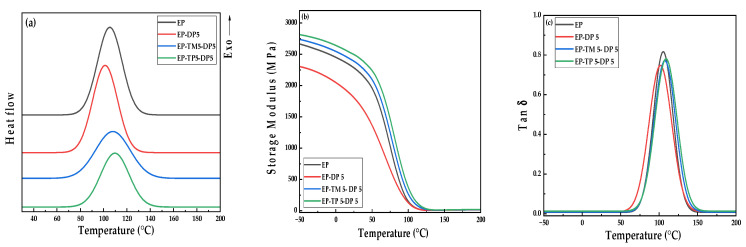
(**a**) DSC thermo-grams; (**b**) storage modulus (G′); and (**c**) tan δ curve as a function of the temperature of epoxy blends.

The glass transition temperature (*T_g_*) is an important parameter for thermosetting resins, and it was measured using DSC. Figure 9a reveals a single *T_g_* for all epoxy blends, indicating good dispersion of additives within the epoxy matrix. As evident in both Figure 9a and Table 7, EP, EP-DP5, EP-TM5-DP5, and EP-TP5-DP5 exhibited *T*_g_ values of 105.33, 101.03, 107.58, and 109.40 °C, respectively. The highest *T_g_* is observed for EP-TP5-DP5, which can be attributed to the increased rigidity imparted by the relatively high number of benzene rings introduced by the phenyl end-caps [71]. On the other hand, incorporating phosphorus-only flame retardants decreased *T_g_* compared to pure EP. This can be explained by their plasticizing effect [72], which weakens interactions between polymer chains and increases free volume within the material [73].

This is consistent with the *T_g_* values measured by DSC, as shown in Figure 9a.

Figure 9b presents the storage modulus measured in the DMA analysis. As shown in Table 7, the EP-TP5-DP5 exhibited the highest storage modulus at 50 °C, reaching 2274.33 MPa. In particular, high storage moduli were also observed across all other temperatures. Figure 9c shows the tan δ curve obtained via DMA, with corresponding glass transition temperatures determined from the peaks. These *T_g_* values (105.43, 102.47, 107.66, and 109.88 °C for EP, EP-DP5, EP-TM5-DP5, and EP-TP5-DP5, respectively) closely match the *T_g_* values measured by DSC in Figure 9a.

### 3.8. Comparative Analysis of the Mechanical Properties of Epoxy Blends

**Figure 10:**
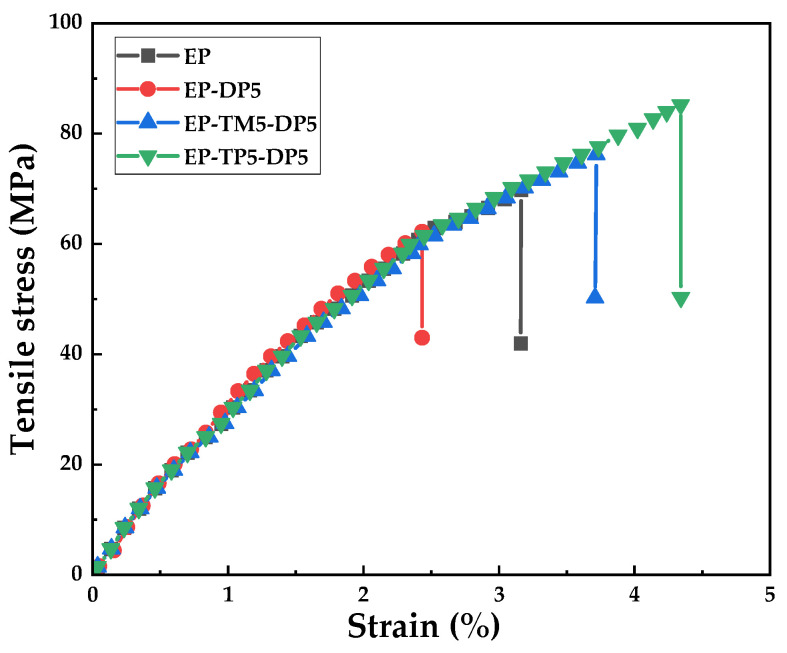
Tensile stress–strain curves of epoxy blends.

Tensile and flexural tests were conducted to evaluate the mechanical properties of epoxy blends. Figure 10 shows the tensile stress–strain curves, and Table 8 summarizes the corresponding tensile and flexural data. Particularly, EP-DP5 with added phosphorus exhibits a significantly reduced crosslinking density in the cured epoxy resin, resulting in a marked decrease in tensile stress and strain. On the other hand, in the case of EP-TM5-DP5 and EP-TP5-DP5 with polysilsesquioxane, the inorganic Si-O-Si core acts as a high-functional anchor, and the flexible organic branches have a reinforcing effect on the DGEBA matrix [74], leading to improved tensile strength and mechanical properties.

Furthermore, EP-TP5-DP5 end-capped with phenyl groups displays superior mechanical properties compared to EP-TM5-DP5 end-capped with methyl groups. The phenyl group is larger and bulkier than the methyl group, affecting the packing and arrangement of the polymer chains, and can increase mechanical properties through steric effects of intermolecular interactions [75,76].

**Figure 11:**
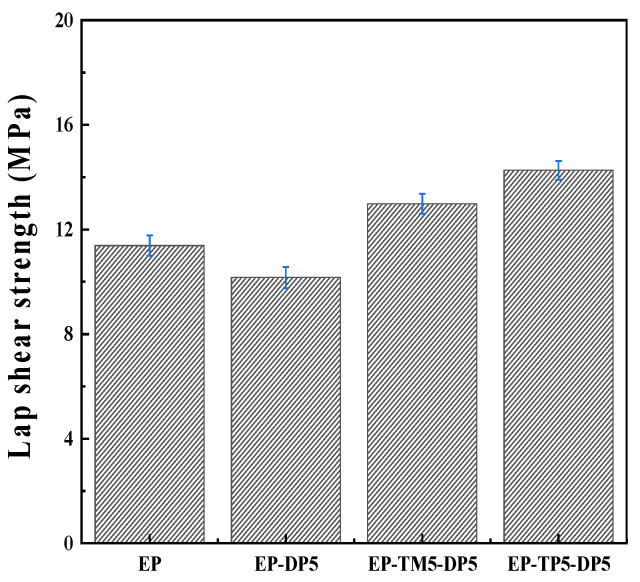
Lap shear strength with epoxy blends: (EP, EP-DP5, EP-TM5-DP5, and EP-TP5-DP5).

Figure 11 compares the lap shear strengths of various epoxy blends, revealing a general decrease in mechanical properties with the addition of phosphorous flame retardants, as reported in the literature [77]. Consistent with expectations, EP-DP5 exhibited a lower lap shear strength than EP. In particular, both EP-TM5-DP5 and EP-TP5-DP5 displayed increased strength compared to EP, as evident in the Figure 11. Among the epoxy blends, EP-TP5-DP5 achieved the highest lap shear strength of 13.26 MPa, representing a 25% improvement over EP. This enhancement can be attributed to the cooperative effect of silicon–sulfur–phosphorus.

### 3.9. Analysis of the VOC Emissions of Epoxy Blends

Table 9 presents the VOC factors for the epoxy blends. These factors include five specific VOCs (benzene, toluene, ethylbenzene, xylene, and styrene) known to be harmful to human health and the environment, as well as the total VOCs (TVOCs), which measure the entire concentration of volatile organic compounds and other organic substances that evaporate into the air, regardless of their potential harm. Table 9 reveals TVOC emissions of 268.45, 230.30, 189.22, and 168.12 µg/m^3^ for EP, EP-DP5, EP-TM5-DP5, and EP-TP5-DP5, respectively. Especially, EP-TM5-DP5 and EP-TP5-DP5, incorporating silicon–sulfur–phosphorus flame retardants, exhibit significantly lower TVOC emissions (<200 µg/m^3^). The observed reduction in VOC emissions can likely be attributed to the high molecular weight of TFLPM and TFLPP. Generally, higher molecular weights correlate with lower vapor pressure, reducing volatility and, consequently, minimizing emissions [78]. These results are consistent with previous findings [79] and suggest that by reducing the potential for vaporization, the release of VOCs during combustion can be minimized, consequently mitigating potential risk in the event of an actual fire.

## 4. Conclusions

This study successfully developed novel ladder-structured polysilsesquioxane flame retardants, end-capped with methyl (CH_3_) and phenyl groups, respectively, via the sol-gel method. Epoxy blends incorporating TFLPM and TFLPP demonstrated superior flame retardancy compared to phosphorus-only flame retardants, which is attributed to the cooperative effects of silicon, phosphorus, and sulfur. The enhancement is ascribed to the cooperative effect of silicon–phosphorus–sulfur. Epoxy blends incorporating silicon–sulfur–phosphorus achieved a UL 94 V-0 rating. The non-flammable gas produced by sulfur decomposition played a crucial role in gas-phase flame retardation by diluting the combustible gas and reducing oxygen concentrations. Among the epoxy blends, those containing TFLPP exhibited the highest LOI value (33.7%) and residual char content according to the LOI Test and TGA analysis. SEM results further indicated that blends containing TFLPP generated a dense, uniform char layer. Epoxy blends enriched with phenyl groups, particularly those containing TFLPP, showed increased stiffness and glass transition temperature (*T_g_*), resulting in a higher storage modulus and a 25% improvement in lap shear strength compared to pure epoxy (EP). Importantly, these blends also demonstrated the lowest volatile organic compound (VOC) emissions.

The developed epoxy blend also provides significant advantages for using 3D printing in additive manufacturing. It improves mechanical strength and flame retardancy while reducing the emission of volatile organic compounds (VOCs). This reduction mitigates potential environmental and health issues that could arise during the thermal curing process of 3D printing.

## Figures and Tables

**Table 1:** Formulations of EP blends.

Blends	Formulation (wt%)				
	EP	TFLPM	TFLPP	DEHMP	m-XDA
EP	90	0	0	0	10
EP-DP5	85	0	0	5	10
EP-TM5-DP5	80	5	0	5	10
EP-TP5-DP5	80	0	5	5	10

**Table 2:** Molecular weights of TFLPM and TFLPP.

Sample	Mn (g/mol)	Mw (g/mol)	PDI (Poly Dispersity Index) [Mw/Mn]
TFLPM	2122	3782	1.78
TFLPP	2340	4682	1.95

**Table 3:** Thermal data obtained from TGA thermograms of TFLPM and TFLPP.

	*T*_dec−5%_ (°C)	*T*_max_ (°C)	A* · K*	IPDT (°C)	Residual Mass (%)
TFLPM	407.38	501.02	0.67	706.31	43.82
TFLPP	429.77	526.17	0.72	725.74	47.53

**Table 4:** Epoxy blends’ thermal stability data obtained from TGA thermograms.

Blends	Atmosphere	*T*_dec−5%_ (°C)	*T*_max1_ (°C)	*T*_max2_ (°C)	A* · K*	IPDT (°C)	Residual Mass (%)
EP	N_2_	305.54	414.77	-	0.57	630.73	18.63
EP-DP5		270.49	372.68	-	0.49	603.47	21.26
EP-TM5-DP5		400.95	487.46	-	0.64	675.52	24.22
EP-TP5-DP5		432.56	504.67	-	0.69	700.83	28.49
EP	Air	293.13	357.41	541.06	0.53	609.54	14.84
EP-DP5		262.54	311.39	484.51	0.44	584.26	17.11
EP-TM5-DP5		383.51	373.77	546.98	0.59	653.61	19.97
EP-TP5-DP5		417.33	481.55	605.76	0.62	688.27	21.56

**Table 5:** The cone calorimeter results of the pure EP and its blends.

Blends	TTI (s)	HRR (KW/m^2^)	THR (MJ/m^2^)
EP	73	868.5	94.3
EP-DP5	77	703.1	76.4
EP-TM5-DP5	85	619.8	66.8
EP-TP5-DP5	91	539.2	56.7

**Table 6:** Elemental analysis of epoxy blends of combustion residual.

Blends	Element Content, wt%					
	Carbon (C)	Oxygen (O)	Nitrogen (N)	Silicon (Si)	Phosphorus (P)	Sulfur (S)
EP	78.71	16.53	4.76	-	-	-
EP-DP5	79.12	15.13	4.31	-	1.44	-
EP-TM5-DP5	81.67	8.61	4.11	3.67	1.46	0.48
EP-TP5-DP5	83.03	6.62	3.92	4.34	1.51	0.58

**Table 7:** Glass transition temperature (*T*_g_) and storage modulus (MPa) of epoxy blends.

Blends	*T*_g_ (DSC) (°C)	*T*_g_ (DMA) (°C)	Storage Modulus (MPa)	
			50 °C	*T*_g_ + 40 °C
EP	105.33	105.43	1947.16	10.49
EP-DP5	101.03	102.47	1394.17	8.91
EP-TM5-DP5	107.58	107.66	2090.30	23.07
EP-TP5-DP5	109.40	109.88	2274.33	31.29

**Table 8:** Mechanical property results of the pure Ep and its blends.

Blends	Tensile Strength (MPa)	Tensile Modulus (GPa)	Flexural Strength (MPa)	Flexural Modulus (GPa)
EP	68.5 ± 1.6	3.16 ± 0.09	97.4 ± 2.7	2.92 ± 0.19
EP-DP5	61.8 ± 1.9	2.42 ± 0.16	89.2 ± 3.4	2.58 ± 0.09
EP-TM5-DP5	75.6 ± 2.8	3.72 ± 0.09	106.8 ± 2.3	3.21 ± 0.15
EP-TP5-DP5	85.3 ± 2.2	4.34 ± 0.19	123.3 ± 1.9	3.58 ± 0.11

**Table 9:** Comparison of VOC emissions for EP, EP-DP5, EP-TM5-DP5, and EP-TP5-DP5.

[µg/m^3^]	EP	EP-DP5	EP-TM5-DP5	EP-TP5-DP5
TVOC	268.45	230.30	189.22	168.12
5VOCs	6.81	6.4	5.9	4.9
Toluene	0.92	0.9	0.83	0.6
Benzene	5.89	5.3	4.1	3.3
Ethylbenzene	0	0	0	0
*m*, *p*-Xylene	0	0	0	0
Styrene	0	0	0	0

## Data Availability

The data presented in this study are available on request from the corresponding author. The data are not publicly available due to privacy or ethical restrictions.

## Supplementary Materials

The following supporting information can be downloaded at: https://www.mdpi.com/article/10.3390/polym16060842/s1. Figure S1: ^1^H NMR Spectra of (a) TFLPM and (b) TFLPP. Figure S2: ^13^C NMR Spectra of (a) TFLPM and (b) TFLPP. Figure S3: MALDI-TOF MS spectra of (a) TFLPM and (b) TFLPP. Table S1: Specific requirements of the UL 94 V test.
