# Supplementary material for: Influence of Thiol-Functionalized Polysilsesquioxane/Phosphorus Flame-Retardant Blends on the Flammability and Thermal, Mechanical, and Volatile Organic Compound (VOC) Emission Properties of Epoxy Resins
Source: Polymers (Basel). 2024 Mar 19;16(6):842. doi: 10.3390/polym16060842 (PMC10975903; doi:10.3390/polym16060842)
Supplement: Supplementary file 1 [file polymers-16-00842-s001.zip › polymers-2907219-supplementary.pdf]

polymers

Supporting Information

Influence of Thiol-Functionalized Polysilsesquioxane/ Phosphorus Flame-Retardant Blends on the Flammability and Thermal, Mechanical, and Volatile Organic Compound (VOC) Emission Properties of Epoxy Resins

Young-Hun Kim [1,2], Jeong Ju Baek [1], Ki Cheol Chang [1], Ho Sun Lim [3], Myung-Seok Choi [4], Won-Gun Koh [2,*] and Gyojic Shin [1,*]

[1] Green Circulation R&D Department, Research Institute of Sustainable Development Technology, Korea Institute of Industrials Technology (KITECH), Yangdaegiro-gil 89, Ipjang-myeon, Cheonan-si 31056, Republic of Korea; kimyh@kitech.re.kr (Y.-H.K.); baekjj@kitech.re.kr (J.J.B.); kcchang@kitech.re.kr (K.C.C.)

[2] Department of Chemical and Biomolecular Engineering, Yonsei University, Yonsei-ro 50, Seodaemun-gu, Seoul 03722, Republic of Korea

[3] Department of Chemical and Biological Engineering, Institute of Advanced Materials and System, Sookmyung Women's University, Seoul 04310, Republic of Korea; limhs@sookmyung.ac.kr

[4] Division of Chemical Engineering, Konkuk University, 120 Neungdong-ro, Gwangjin-gu, Seoul 05029, Republic of Korea; mchoi@konkuk.ac.kr

* Correspondence: wongun@yonsei.ac.kr (W.-G.K.); gyshin@kitech.re.kr (G.S.)

Figure S1a,b presents similar ^{1}H NMR spectra for synthesized TFLPM and TFLPP, confirming the successful grafting of mercaptopropyl arms through characteristic peaks. Four signals in the spectra correspond to the mercaptopropyl chain repeat unit: methylene protons (a, b, c) and the thiol proton (d). Additionally, the TFLPM spectrum exhibited peaks at δ 0.07–0.12 ppm, attributed to the Si−CH_3 group. The TFLPP spectrum exhibited peaks at δ 7.38–7.56 ppm, attributed to the Si−PH group. Calibration with $CDCl_3$ solvent yielded a peak chemical shift [δ] of 7.3 ppm.

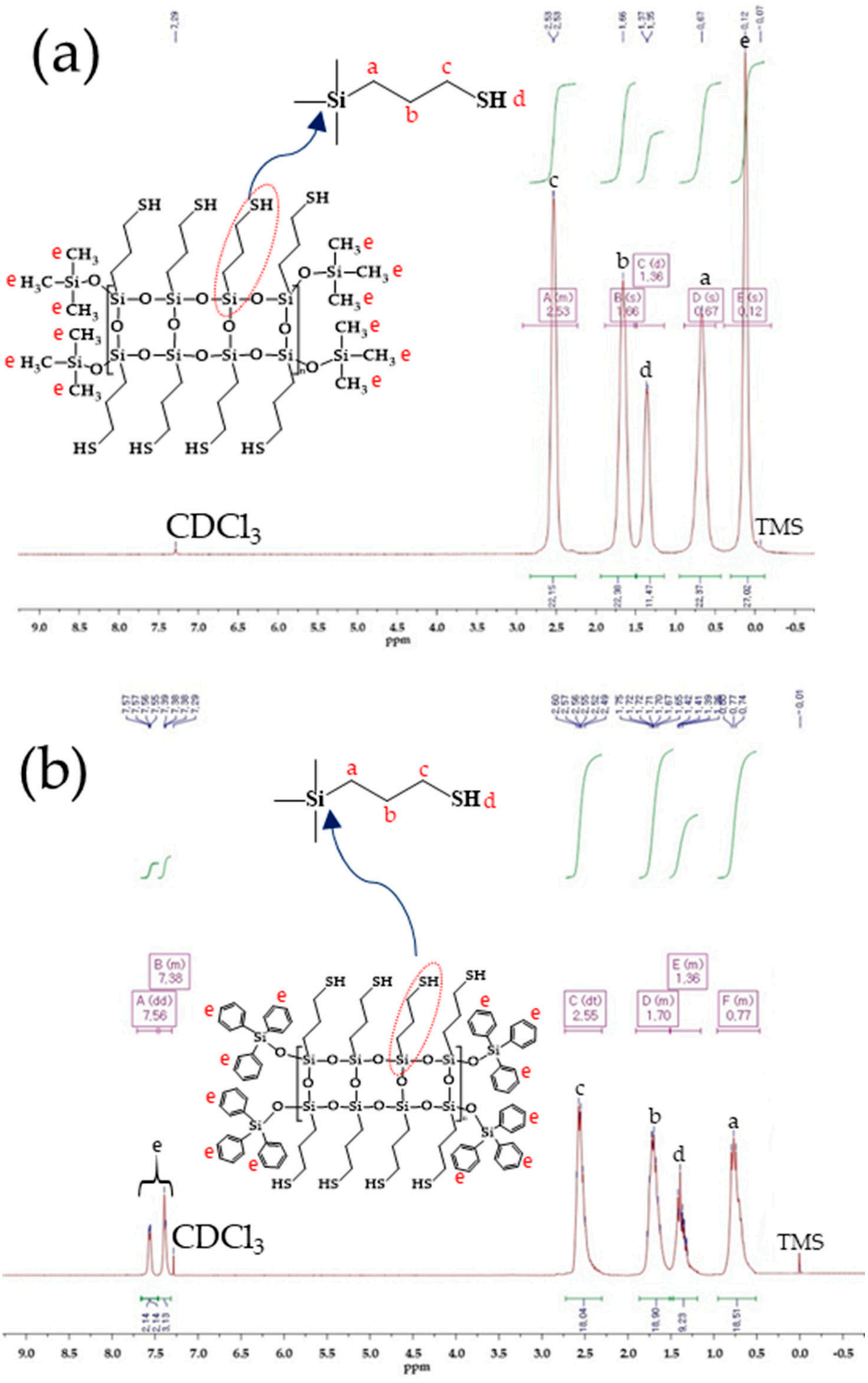

Figure S1. ^{1}H NMR Spectra of (**a**) TFLPM and (**b**) TFLPP.

Consistent with the ^{1}H NMR findings, the ^{13}C NMR spectra of TFLPM and TFLPP in Figure S2a,b exhibit peaks confirming the presence of grafted mercaptopropyl arms. In both TFLPM and TFLPP, two signals corresponding to methylene carbons a and b, c were assigned to the mercaptopropyl repeating unit. The TFLPM spectrum exhibited a peak at δ 1.44 ppm due to the Si–CH_3 group, while TFLPP presented peaks at δ 127.82 (ortho–C), δ 130.28 (para–C), and δ 134.06 ppm (meta–C) arising from the presence of phenyl groups. The 77 ppm triplet observed in the ^{13}C NMR spectrum indicates coupling between the carbon of the solvent $CDCl_3$and deuterium (I = 1).

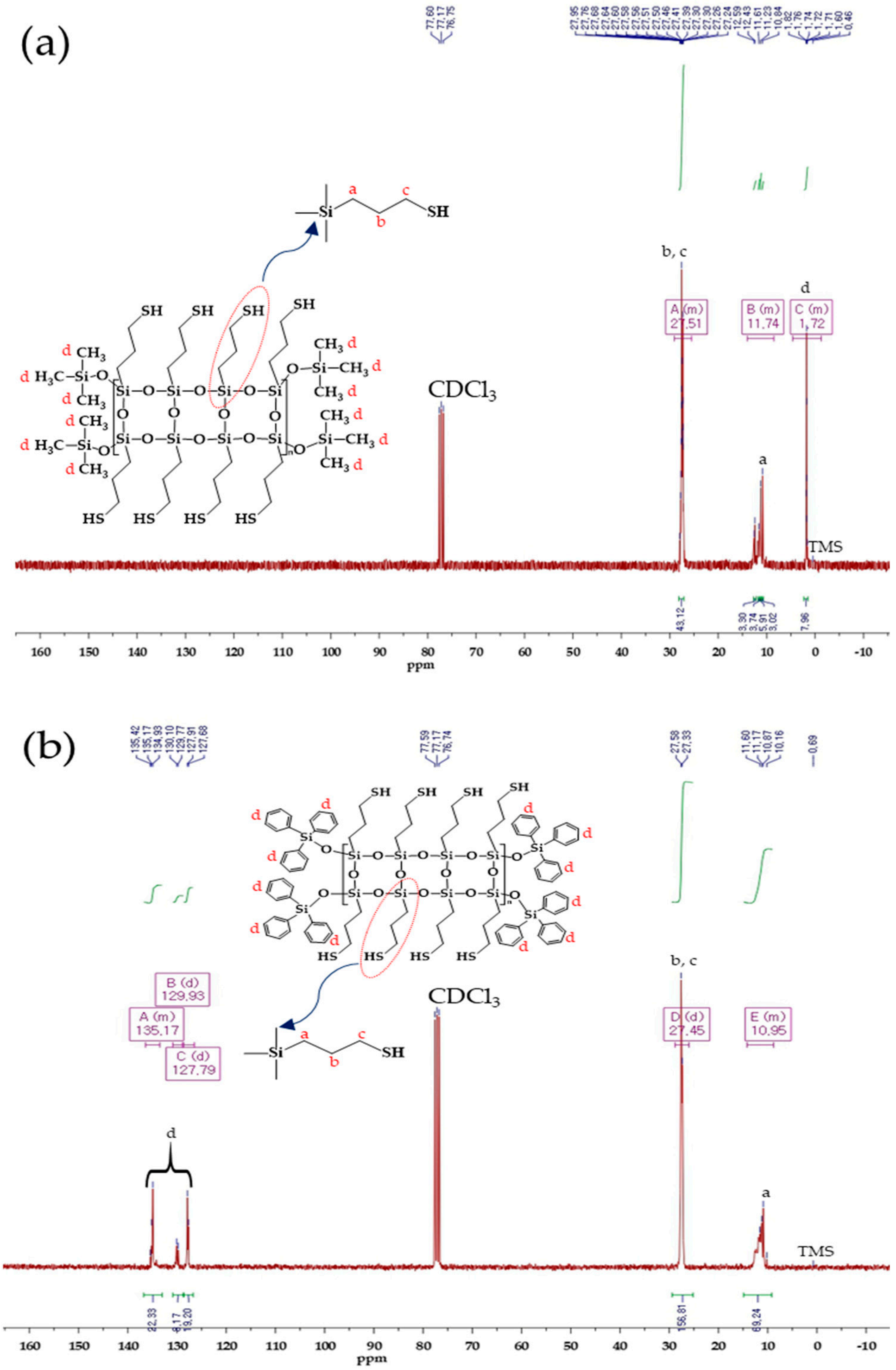

Figure S2. ^{13}C NMR Spectra of (**a**) TFLPM and (**b**) TFLPP.

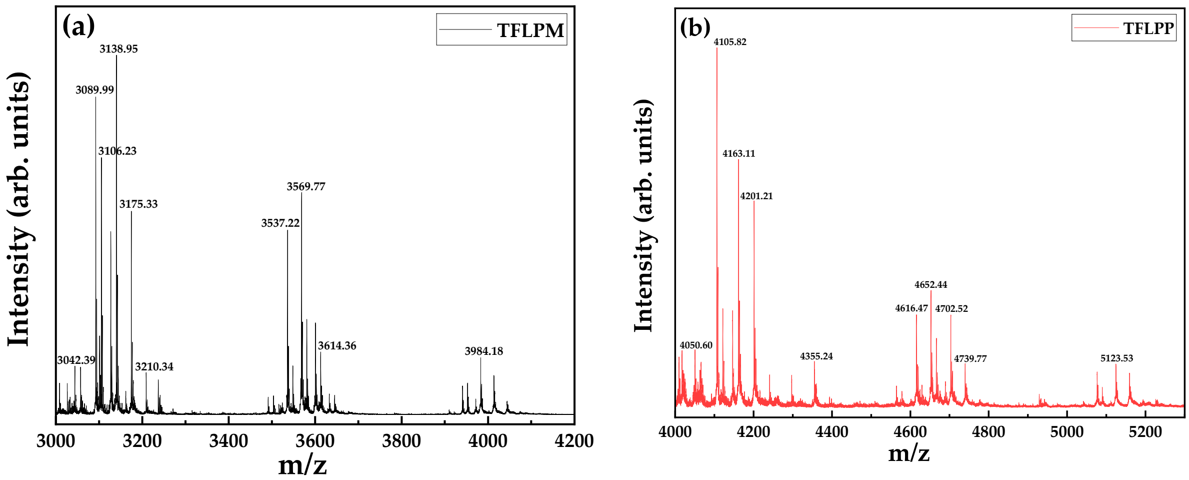

Figure S3. MALDI-TOF MS spectra of (**a**) TFLPM and (**b**) TFLPP.

Table S1. Specific requirements of the UL 94 V test

Criteria	Category		
	**V-0**	**V-1**	**V-2**
**Burning time of each individual test specimen**	≤10 s	≤30 s	≤30 s
**Total after flame time for any condition set [5 flame applications]**	≤50 s	≤250 s	≤250 s
**Individual test specimen after flame plus afterglow time after the second flame application**	≤30 s	≤60 s	≤60 s
**Dripping of burning specimens [ignition of cotton batting]**	No	No	Yes
**Combustion up to holding clamp [specimens completely burned]**	No	No	No
